# Supplementary material for: Detection and Measurement of Hypopyon on Slit Lamp Examination Versus Anterior Segment Optical Coherence Tomography
Source: Bioengineering (Basel). 2026 May 19;13(5):582. doi: 10.3390/bioengineering13050582 (PMC13203708; doi:10.3390/bioengineering13050582)
Supplement: Supplementary file 1 [file bioengineering-13-00582-s001.zip › bioengineering-4296441-supplementary.pdf]

Supplementary Figure S1. Composite images used for ASOCT hypopyon grading.

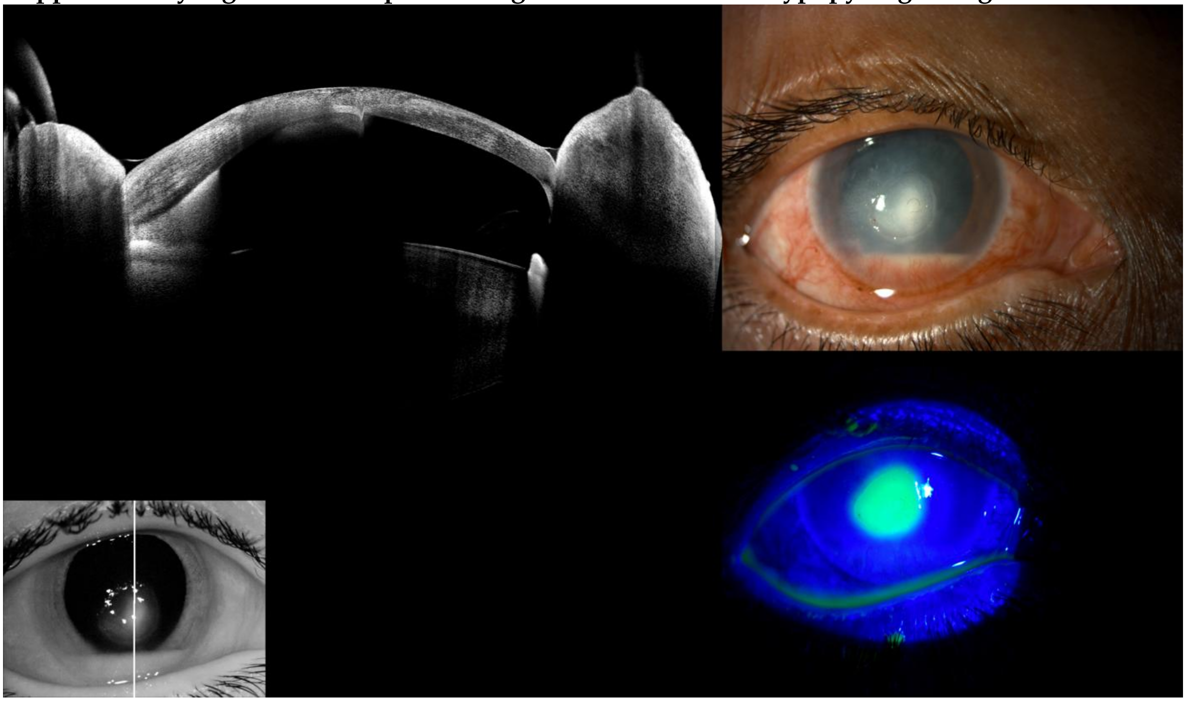

Supplementary Figure S2. Eligibility criteria for assessment of ASOCT detection of hypopyon compared to slit lamp examination, intra-grader repeatability for hypopyon detection, and hypopyon height measurement.

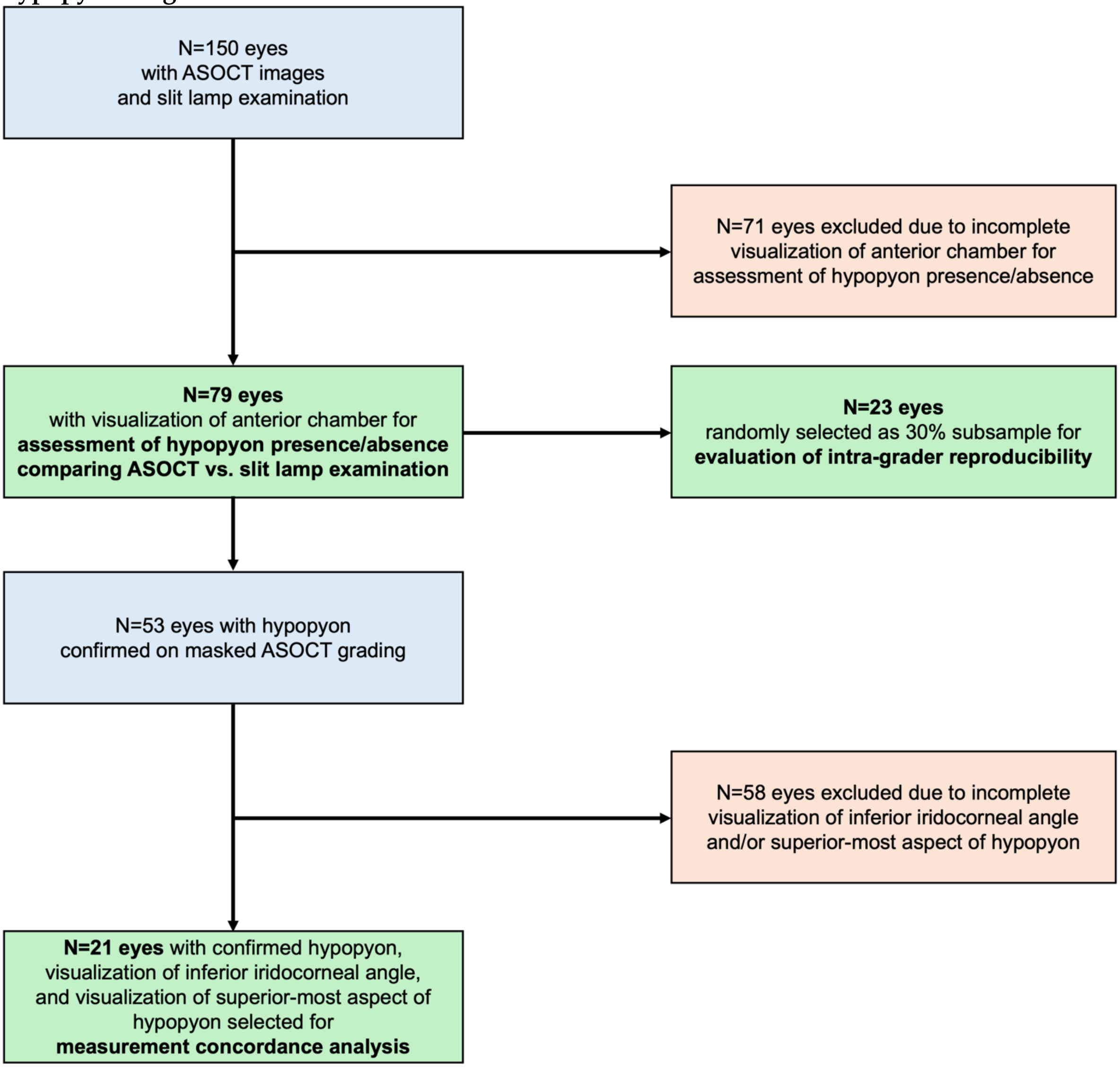

The 58 eyes excluded from hypopyon height measurement analysis included 26 eyes without hypopyon and 32 eyes with hypopyon but with incomplete visualization of the inferior iridocorneal angle and/or the superior-most hypopyon margin.

Supplementary Table S1: Intra-grader, inter-grader and diagnostic concordance for hypopyon detection comparing ASOCT-only grading and slit lamp examination.

Agreement	Hypopyon
**Intra-grader agreement, Grader 1[a]**	
Hypopyon present in session 1, n / N (%)	18 / 23 (78.3)
Hypopyon present in session 2, n / N (%)	18 / 23 (78.3)
Percent agreement (95% CI)	100.0 (100.0-100.0)
Kappa statistic (95% CI)[b]	1.0 (1.0-1.0)
**Intra-grader agreement, Grader 2[a]**	
Hypopyon present in session 1, n / N (%)	16 / 23 (69.6)
Hypopyon present in session 2, n / N (%)	16 / 23 (69.6)
Percent agreement (95% CI)	91.3 (78.3-100.0)
Kappa statistic (95% CI)[b]	0.79 (0.49-1.00)
**Inter-grader agreement:**<br>**Grader 1 vs. Grader 2[b]**	
Grader 1: Hypopyon present, n / N (%)	54 / 79 (68.4)
Grader 2: Hypopyon present, n / N (%)	53 / 79 (67.1)
Overall percent agreement	92.4 (87.3–97.5)
Kappa statistic	0.83 (0.69–0.95)
**Diagnostic Concordance Parameters[c]**	
Hypopyon noted on masked ASOCT grading, N (%)	55/79 (69.6)
Hypopyon noted on in-person slit lamp examination, N (%)	45/79 (57.0)
Percent agreement	84.8 (75.9–92.4)
Kappa statistic	0.68 (0.51–0.83)
Sensitivity	80.0 (68.4–90.0)
Specificity	95.8 (87.0–100.0)
Positive predictive value	97.8 (92.7–100.0)
Negative predictive value	67.6 (50.0–83.0)

[a] Intra-grader agreement analysis was restricted to N=23 eyes comprising a randomly selected 30% subsample of N=79 eyes that had been graded for presence or absence of hypopyon. These 23 eyes were independently evaluated by two graders across two different grading sessions.
[b] Inter-grader agreement analysis was restricted to N=79 eyes with adequate visualization of the anterior chamber on ASOCT images without obscuration from eyelids.
[c] When calculating sensitivity, specificity, positive predictive value, and negative predictive value, in-person slit lamp examination was compared against ASOCT consensus grading, with the latter assessment used as the reference diagnostic standard.
Values in parentheses are 95% percentile-based confidence intervals from bootstrapping (1,000 resamples).
Abbreviations: ASOCT = anterior segment optical coherence tomography; CI = confidence interval; N = number.
Kappa statistic interpretation: poor (<0.00), slight (0.00–0.20), fair (0.21–0.40), moderate (0.41–0.60), substantial (0.61–0.80), almost perfect (0.81–1.00).

Supplementary Table S2. Hypopyon detection comparing ASOCT and slit lamp examination, by clinical subgroup.

Clinical Characteristic	Agreement N (%)[a]	Disagreement N (%)[a]	Unadjusted OR (95% CI)[b]	P value
**Visual acuity**				
LogMAR <1.0	8 (11.6)	2 (20.0)	Reference	-
LogMAR ≥1.0	61 (88.4)	8 (80.0)	0.53 (0.08–5.98)	0.74
**Infection type**				
Fungal only	34 (49.3)	6 (60.0)	Reference	-
Bacterial only	27 (39.1)	3 (30.0)	0.63 (0.09-3.30)	0.81
Polymicrobial	8 (11.6)	1 (10.0)	0.71 (0.14-7.35)	1.00
**Infiltrate diameter**				
Not applicable	0 (0.0)	1 (10.0)	Reference	-
0 to <2 mm	7 (10.1)	0 (0.0)	0.14 (0.00-5.57)[c]	*0.25*
2 to <6 mm	51 (73.9)	8 (80.0)	0.18 (0.00-6.88)[c]	*0.3*
≥6 mm	11 (15.9)	1 (10.0)	0.18 (0.00- 7.09)[c]	*0.31*
**Infiltrate depth**				
Anterior 1/3 stroma[d]	64 (92.8)	7 (70.0)	0.19 (0.03-1.47)	0.12
Middle 1/3 stroma[d]	48 (69.6)	7 (70.0)	1.02 (0.21-6.71)	1.00
Posterior 1/3 stroma[d]	21 (30.4)	2 (20.0)	0.58 (0.06-3.24)	0.79
**Stromal thinning present**				
No	46 (66.7)	8 (80.0)	Reference	
Yes	23 (33.3)	2 (20.0)	0.50 (0.05-2.82)	0.65
**Infiltrate within 2 mm of limbus**				
No	63 (91.3)	10 (100.0)	Reference	
Yes	6 (8.7)	0 (0.0)	0.81 (0.00-6.24)[c]	*0.86*
**Endothelial plaque present**				
No	65 (94.2)	9 (90.0)	Reference	
Yes	4 (5.8)	1 (10.0)	1.79 (0.03– 21.03)	1.00

[a] N (%) represents the number and percentage of eyes within each group. Odds ratios and 95% confidence intervals were estimated using univariable exact logistic regression.

[b] Unadjusted OR of disagreement from univariable exact logistic regression.

[c] Odds ratio estimates are median unbiased estimates derived from the exact conditional distribution, used because zero cell counts in one or more categories resulted in complete separation, rendering maximum likelihood estimates undefined.

[d] Reference for infiltrate depth = absence of involvement in respective categories.

Abbreviations: ASOCT = anterior segment optical coherence tomography; CI = confidence interval; logMAR = logarithm of minimum angle of resolution; N = number; OR = odds ratio for disagreement between ASOCT and slit lamp examination.
